# Prediction of tide level based on variable weight combination of LightGBM and CNN-BiGRU model

**DOI:** 10.1038/s41598-022-26213-y

**Published:** 2023-01-02

**Authors:** Ye Su, Xuchu Jiang

**Affiliations:** School of Statistics and Mathematics, Zhongnan University of Economics and Law, Wuhan, People’s Republic of China

**Keywords:** Ecology, Environmental sciences, Hydrology, Limnology, Natural hazards, Ocean sciences, Engineering, Mathematics and computing

## Abstract

Accurate tide level prediction is crucial to human activities in coastal areas. Many practical applications show that compared with traditional harmonic analysis, long short-term memory (LSTM), gated recurrent units (GRUs) and other neural networks, along with ensemble learning models, such as light gradient boosting machine (LightGBM) and eXtreme gradient boosting (XGBoost), can achieve extremely high prediction accuracy in relatively stationary time series. Therefore, this paper proposes a variable weight combination model based on LightGBM and CNN-BiGRU with relevant research. It uses the variable weight combination method to weight and synthesize the prediction results of the two base models so that the combination model has a stronger ability to capture time series features and fits the data well. The experimental results show that in contrast to the base model LightGBM, the RMSE value and MAE value of the combination model are reduced by 43.2% and 44.7%, respectively; in contrast to the base model CNN-BiGRU, the RMSE value and MAE value of the combination model are reduced by 35.3% and 39.1%, respectively. This means that the variable weight combination model can greatly improve the accuracy of tide level prediction. In addition, we use tidal data from different geographical environments to further verify the good universality of the model. This study provides a new idea and method for tide prediction.

## Introduction

Tide refers to seawater moving periodically under the influence of celestial bodies’ gravitation (moon and sun). The change in the water level caused by tides has a profound impact on engineering construction, the ecological environment and people's development and utilization of marine resources in coastal areas. For a long time, tide forecasting not only provided safety guarantees for port operations, shipping traffic and coastal protection but also provided important information for offshore aquaculture and the prevention of meteorological disasters. Currently, while promoting the use of clean energy such as tidal energy, we find that accurate tidal level prediction is not only directly related to the power generation efficiency of tidal power stations but also the key to formulating a reasonable dispatching scheme for tidal power stations. Therefore, the accurate prediction of the tide level is of great significance to both human activities and the construction and development of the ocean in coastal areas.

To carry out tide prediction, many domestic and foreign scholars have established models to describe and study tide observation data. Harmonic analysis is a classical tidal data analysis method. Based on the assumption that the amplitude and lag angle of each component tide are constant, we can calculate the harmonic constants and then predict the component tide. After Darwin^1^ put forward the equilibrium tide theory, Doodson^2–4^ used the least squares method to process tide observation data and determine the harmonic analysis constant, but this method is only applicable to the equilibrium tide. To overcome the shortcomings of the harmonic analysis method and improve the prediction accuracy, scholars have been studying and improving it for a hundred years. Kukulka et al.^5,6^ introduced nonstationary external force forcing (river runoff and open sea tide) into the basic function of harmonic analysis and proposed a nonstationary fluvial tide model to make the harmonic analysis applicable to nonequilibrium tides such as estuarine tides. Jin et al.^7^ proposed the nonstationary tidal harmonic analysis method EHA (enhanced harmonic analysis), which turned the amplitude and epoch of partial tide (both are constants) in harmonic analysis into a function varying with time, used the independent point scheme^8–11^ and cubic spline interpolation method to optimize the solution of each component tide’s amplitude and delay angle, and generalized the harmonic analysis to the analysis of internal tidal velocity. With the development of computer science and technology, Matte^12^, Pan^13^ and others developed NS_TIDE, S_TIDE and other harmonic analysis toolkits based on the above models. Now, the S_Tide toolkit can be applied to the analysis and prediction of all types of tides in theory. Although the harmonic analysis method can effectively predict tides after continuous development, there will still be large errors in the prediction of tides if we only use the harmonic analysis method due to the influence of environmental factors such as air pressure, wind force and terrain in reality^14^.

With the continuous development of machine-learning theory, analysis and prediction models such as neural networks are emerging, which not only provide a new method for the simulation, prediction and control of complex systems but also bring a new method for tide prediction research. Tsai et al.^15^ first used a backpropagation (BP) neural network to predict full-day and half-day tides and then extended the research to multiple sites^16^. Zhang et al.^17^ used an adaptive particle swarm optimization algorithm to improve a BP neural network for tide prediction. Later, Zhang et al.^18^ proposed the gray GMDH model combined with harmonic analysis to predict astronomical tides and nonastronomical tides and achieved good results. Zhu et al.^19^ used bidirectional long short-term memory (BiLSTM) networks to predict the tide level of the Isabel port. The simulation results show that the prediction result of the BiLSTM network is better than that of the unidirectional long short-term memory (LSTM) networks. Yang et al.^20^ established an LSTM model and used nearly 21 years of data to predict the tide level of 17 ports in Taiwan. By comparing various models, it is found that the LSTM model has higher stability and stronger prediction ability. Huang et al.^21^ noted that only using recurrent neural network models^22^ such as LSTM cannot effectively mine the local features and potential relationships of tide level sequences, so they use a one-dimensional convolutional neural network (CNN) to extract the local features in tide level sequences, which improves the prediction accuracy of the model.

At the same time, we note that in addition to neural networks, models based on ensemble learning, such as light gradient boosting machine^23^ (LightGBM) and eXtreme gradient boosting^24^ (XGBoost), are also widely used in time series prediction^25–27^, which can achieve good results in predicting relatively stationary time series. Moreover, some researchers have also tried to improve the accuracy of time series prediction by combining several single machine-learning models. Zhang et al.^28^ constructed a prediction model based on the combination of wavelet noise reduction and LSTM, which improved the accuracy of coal mine gas concentration prediction. Han et al.^29^ constructed a gas concentration residual correction model based on a Markov model and a gray neural network, and the combination model had better results than the single model. However, most combination models put the first prediction results into another model for secondary prediction or simply add the prediction results of the two models to obtain the average value, which does not improve the prediction accuracy of the model.

To further improve the accuracy of tide prediction and overcome the shortcomings of the above research, we propose a variable weight combination model based on LightGBM and CNN-BiGRU, which will be simply written as LightGBM-CNN-BiGRU (combination model). The combination model is integrated with the LightGBM and CNN-BiGRU models to predict the tide level in a short time. Aiming at the disadvantages that the traditional LSTM network generally has a slow training speed and is weak in extracting sequence features from tidal observations, we add a local feature preextraction module (one-dimensional CNN) before the recurrent neural network to preextract the local features of the tide level sequence^21^. Through many experiments, we found that BiGRU enjoys a better prediction ability than BiLSTM, so we use the BiGRU network rather than the BiLSTM network for subsequent prediction tasks. The LightGBM-CNN-BiGRU (combination model) will carry out parallel prediction through the CNN-BiGRU network and LightGBM model and then generate the most accurate tide level prediction through the variable weight combination method.

The experimental results show that compared with the base models (LightGBM, BiGRU and BiLSTM) and another variable weight combination model based on LightGBM and BiGRU (LightGBM-BiGRU (combination model)), the model we propose (LightGBM-CNN-BiGRU (combination model)) effectively improves the accuracy of tide level prediction and thus can provide more reliable data guarantees for marine construction and development. Our experiment also shows that the combination model can reduce the absolute error to approximately 0.03 m with tide observations of only one quarter, and its architecture is not complicated, so it deserves to be used in practice.

## Data sources

**Figure 1:**
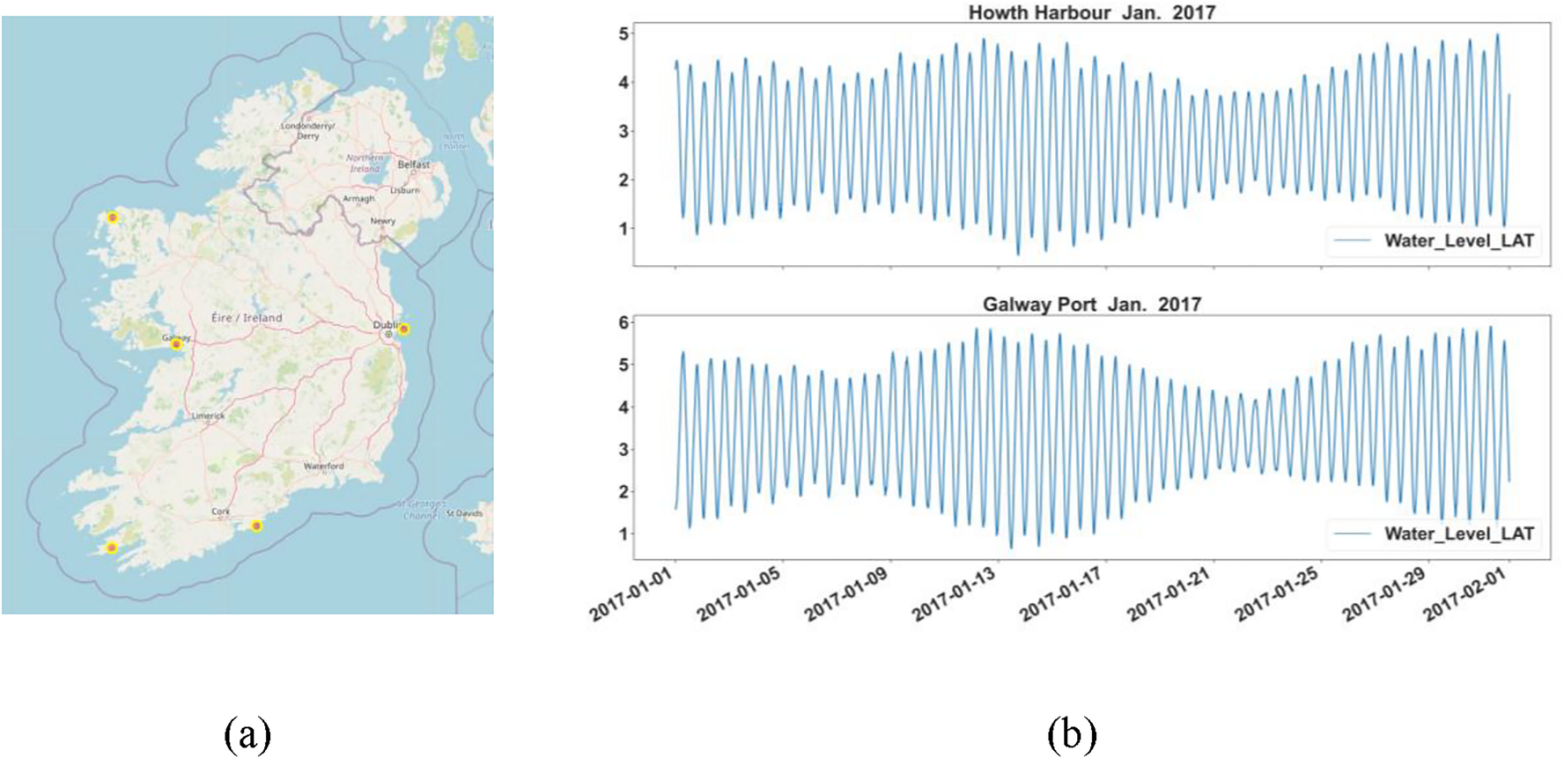
Location distribution of each site and partial tidal observation sequences.

To carry out effective tide prediction, most coastal countries will set up tide measurement stations in their main ports to collect enough tidal observations. The data we used in this study come from the real-time tide monitoring network INTGN (the Irish National tide gauge network), which is operated by the Irish Marine Institute. INTGN consists of 20 tide measuring stations. We select the observation data of Howth Harbor station in Dublin, the capital of Ireland, to build our tide prediction model. In addition, we also selected data from four other sites along the Irish coastline to analyze the generality and generalization ability of our model. The location distribution of the five stations is shown in Fig. 1a. All of the experimental data were subjected to quality control. The time span is from January 1, 2017, to March 31, 2017, and the recording interval is 6 min. In Fig. 1b, we show some subsequences of the tidal observation sequence. Table 1 briefly describes the sequence information of the five sites.

**Table 1:** Data description of each site.

Site	Latitude (°)	Longitude (°)	Mean (m)	SD (m)	Min (m)	Max (m)
Howth Harbor	53.3915	− 6.0683	2.887	1.115	0.447	5.177
Ballycotton Harbor	51.8278	− 8.0007	2.456	1.077	0.277	4.848
Ballyglass Harbor	54.2536	− 9.8928	2.357	0.931	0.164	4.625
Castletownbere Port	51.6496	− 9.9034	2.316	0.875	0.481	4.301
Galway Port	53.2690	− 9.0480	3.458	1.242	0.590	6.160

### Treatment of missing values

After simple statistics, only a small number of tidal observations are missing. As shown in Fig. 1b, the hourly change in the offshore tide level is slow. Moreover, the dataset also enjoys a high frequency of tide level recording, so even if some time points are missing, their values should be close to the observations at adjacent times. For the missing data in some time steps, we use quadratic interpolation.

Given a piece of time series, construct a curve passing through those data points and use the curve to predict values of the curve at other time points. We want to interpolate a curve through the three time points shown below. Since we have three time points, the simplest curve that passes through all three of those points will be a quadratic polynomial. Our job is to compute that polynomial and use it to predict the value of y at other time points between ${t}_{0}$ and ${t}_{2}$.

**Table:** 

Time points	${t}_{n}$	${y}_{n}$
0	${t}_{0}$	${y}_{0}$
1	${t}_{1}$	${y}_{1}$
2	${t}_{2}$	${y}_{2}$

The first method to compute the polynomial starts with the assumption that the polynomial takes the form

1 $$\begin{array}{c}p\left(x\right)=a{x}^{2}+bx+c\end{array}$$

Substituting the three time points into this curve gives a set of three equations in three unknowns.

2 $$\begin{array}{c}\left\{\begin{array}{c}{y}_{0}=a{t}_{o}^{2}+b{t}_{0}+c\\ {y}_{1}=a{t}_{1}^{2}+b{t}_{1}+c\\ {y}_{2}=a{t}_{2}^{2}+b{t}_{2}+c\end{array}\right.\end{array}$$

These equations can be rewritten as a matrix–vector system.

3 $$\begin{array}{c}\left[\begin{array}{c}{y}_{0}\\ {y}_{1}\\ {y}_{2}\end{array}\right]=\left[\begin{array}{ccc}{t}_{o}^{2}& {t}_{0}& 1\\ {t}_{1}^{2}& {t}_{1}& 1\\ {t}_{1}^{2}& {t}_{2}& 1\end{array}\right]\left[\begin{array}{c}a\\ b\\ c\end{array}\right]\end{array}$$

By computing the inverse of the matrix, we can then solve this system for the coefficients of the polynomial.

4 $$\begin{array}{c}\left[\begin{array}{c}a\\ b\\ c\end{array}\right]={\left[\begin{array}{ccc}{t}_{o}^{2}& {t}_{0}& 1\\ {t}_{1}^{2}& {t}_{1}& 1\\ {t}_{1}^{2}& {t}_{2}& 1\end{array}\right]}^{-1}\left[\begin{array}{c}{y}_{0}\\ {y}_{1}\\ {y}_{2}\end{array}\right]\end{array}$$

### Data standardization

We use neural networks when constructing the combination model, and the normalized data can reduce the computational complexity and accelerate the convergence of the network. Therefore, it is necessary to normalize the data. We normalize the tidal observation sequences by applying the Min–Max normalization. The normalization formula is:

5 $$\begin{array}{c}{x}^{*}=\frac{x-{x}_{min}}{{x}_{max}-{x}_{min}}\end{array}$$

where ${x}^{*}$ is the data value after standardization and ${x}_{max}$ and ${x}_{min}$ are the maximum and minimum values in the sample sequences, respectively.

### Problem formulation

#### Notation

Let $\mathcal{X},\mathcal{Y}\subseteq {\mathbb{R}}$ be sets. For a set $\mathcal{X}$, let ${\mathcal{X}}^{*}:={\bigcup }_{\mathrm{T}\in {\mathbb{N}}}{\mathcal{X}}^{T}$ be finite sequences in $\mathcal{X}$. For $x\in {\mathcal{X}}^{T}\subseteq {\mathcal{X}}^{*}(T\in {\mathbb{N}})$, denote by $|x|:=T$ the length of $x$. For $x$ and $y$ being vectors, we denote by $\mathcal{X}$ the predictor space and by $\mathcal{Y}$ the target space.

#### The time series forecasting problem

Time series forecasting, in terms of a supervised learning problem, can be formulated as follows:

Given a set $\mathcal{X}:={\left({\mathbb{R}}^{M}\times {\mathbb{R}}^{L}\right)}^{*}$ and a set $\mathcal{Y}:={\mathbb{R}}^{h\times L}$, with $M,L,h\in {\mathbb{N}}$, a sample $\mathcal{D}\in (\mathcal{X}\times \mathcal{Y}{)}^{*}$ from an unknown distribution $p$ and a loss function $\ell:\mathcal{Y}\times \mathcal{Y}\to {\mathbb{R}}$, find a function $\widehat{y}:\mathcal{X}\to \mathcal{Y}$ called the model that minimizes the expected loss:

6 $$\begin{array}{c}min {\mathbb{E}}_{\left(\left(x,y\right),{y}^{\mathrm{^{\prime}}}\sim p\right)}\left(\ell\left({y}^{\mathrm{^{\prime}}},\widehat{y}\left(x,y\right)\right)\right)\end{array}$$

The predictors in Eq. (6) consist of sequences of vector pairs, $(x,y)$, including the target vector $y$ and the covariate vector $x$ from past time steps, whereas the target sequence is denoted by ${y}^{\mathrm{^{\prime}}}$. In this paper, we focus on the univariate time series forecasting problem for which there is only a single channel, $L = 1$, and no additional covariates are considered, i.e., $M = 0$, such that the predictors consist only of sequences of target channel vectors, $y$.

### Building input data

**Figure 2:**
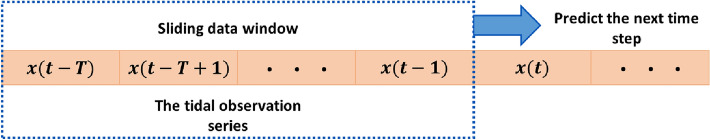
Schematic diagram of the sliding data window.

On the basis of the problem formulation in “Problem formulation”, we transform the problem (to predict the tide level accurately in a short time) into a supervised learning task in machine learning. Therefore, we build our input data as predictors by applying the sliding data window, that is, using the tidal observations in $T$ time steps to predict the tide level in the next time step. The construction principle of the input data is shown in Fig. 2.

## Materials and methods

### LightGBM

Before explaining LightGBM^23^, it is necessary to introduce XGBoost^24^, which is also based on the gradient boosting decision tree (GBDT) algorithm^30^. XGBoost integrates multiple classification and regression trees (CART) to compensate for the lack of prediction accuracy of a single CART. It is an improved boosting algorithm based on GBDT, which is popular due to its high processing speed, high regression accuracy and ability to process large-scale data^31^. However, XGBoost uses a presorted algorithm to find data segmentation points, which takes up considerable memory in the calculation and seriously affects cache optimization.

**Figure 3:**
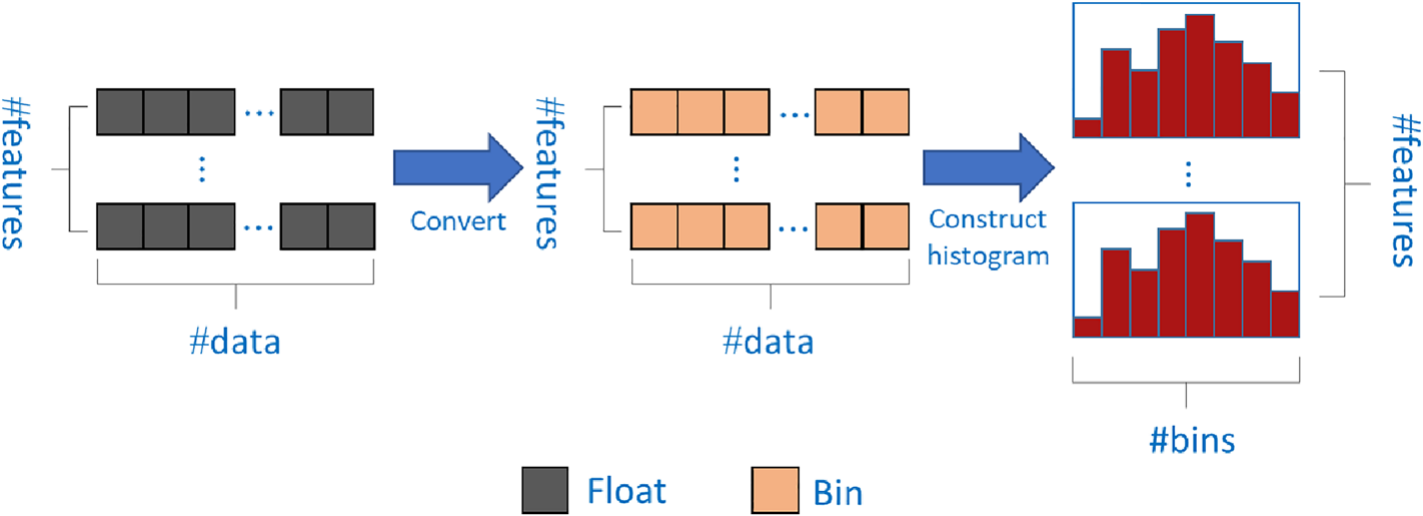
Histogram algorithm.

LightGBM is improved based on XGBoost. It uses a histogram algorithm to find the best data segmentation point, which occupies less memory and has a lower complexity of data segmentation. The flow of the histogram algorithm to find the optimal segmentation point is shown in Fig. 3.

**Figure 4:**
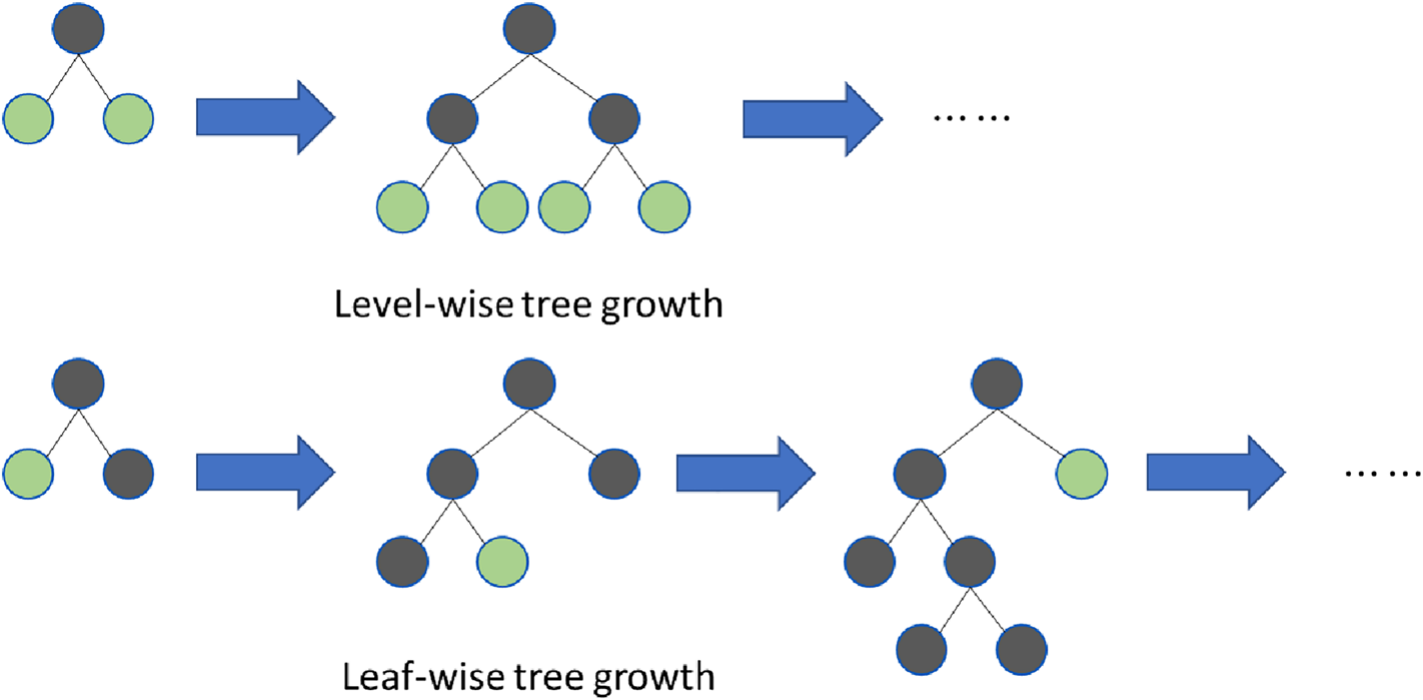
Two GBDT growth strategies.

Moreover, LightGBM abandons the levelwise decision tree growth strategy used by most GBDT tools and uses the leafwise algorithm with depth limitations. This leaf-by-leaf growth strategy can reduce more errors and obtain better accuracy. Decision trees in boosting algorithms may grow too deep while training, leading to model overfitting. Therefore, LightGBM adds a maximum depth limit to the leafwise growth strategy to prevent this from happening and maintains its high computational efficiency. To summarize, LightGBM can be better and faster used in industrial practice and is also very suitable as the base model in our tide level prediction task. The layer-by-layer growth strategy and leaf-by-leaf growth strategy are shown in Fig. 4.

### CNN-BiGRU

#### Convolutional neural network

**Figure 5:**
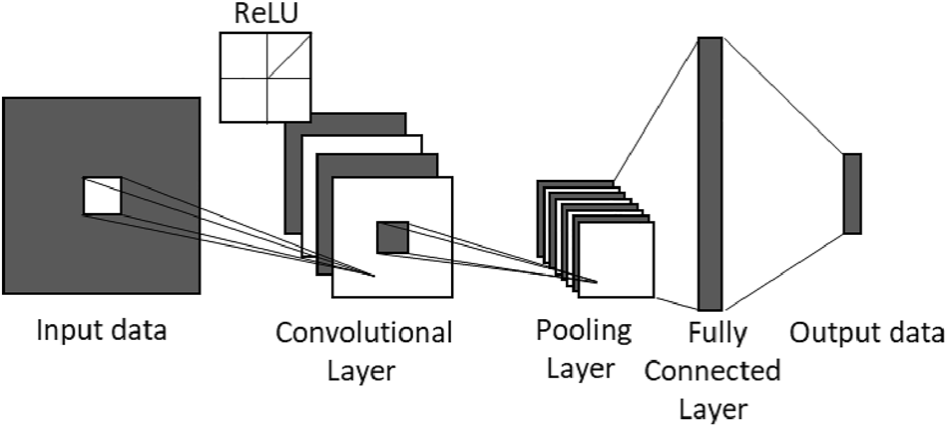
Schematic diagram of a convolutional neural network.

A convolutional neural network (CNN) is a deep feedforward neural network with the characteristics of local connection and weight sharing. It was first used in the field of computer vision and achieved great success^32,33^. In recent years, CNNs have also been widely used in time series processing. For example, Bai et al.^34^ proposed a temporal convolutional network (TCN) based on a convolutional neural network and residual connections, which is not worse than recurrent neural networks such as LSTM in some time series analysis tasks. At present, a convolutional neural network is generally composed of convolution layers, pooling layers and a fully connected layer. Its network structure is shown in Fig. 5. The pooling layer is usually added after the convolution layers. The maximum pooling layer can retain the strong features in the data after the convolution operation, eliminate the weak features to reduce the number of parameters in a network and avoid overfitting of the model.

#### Bidirectional GRU

In previous attempts at tide level prediction by scholars, bidirectional long short-term memory networks^35^ have achieved good prediction results. However, in our subsequent experiments, the bidirectional gated recurrent unit achieved higher prediction accuracy than BiLSTM, so we used the BiGRU network for subsequent prediction tasks.

The GRU network^36^ adds a gating mechanism to control information updating in a recurrent neural network. Different from the mechanism in LSTM, GRU consists of only two gates called the update gate ${z}_{t}$ and the reset door ${r}_{t}$.

**Figure 6:**
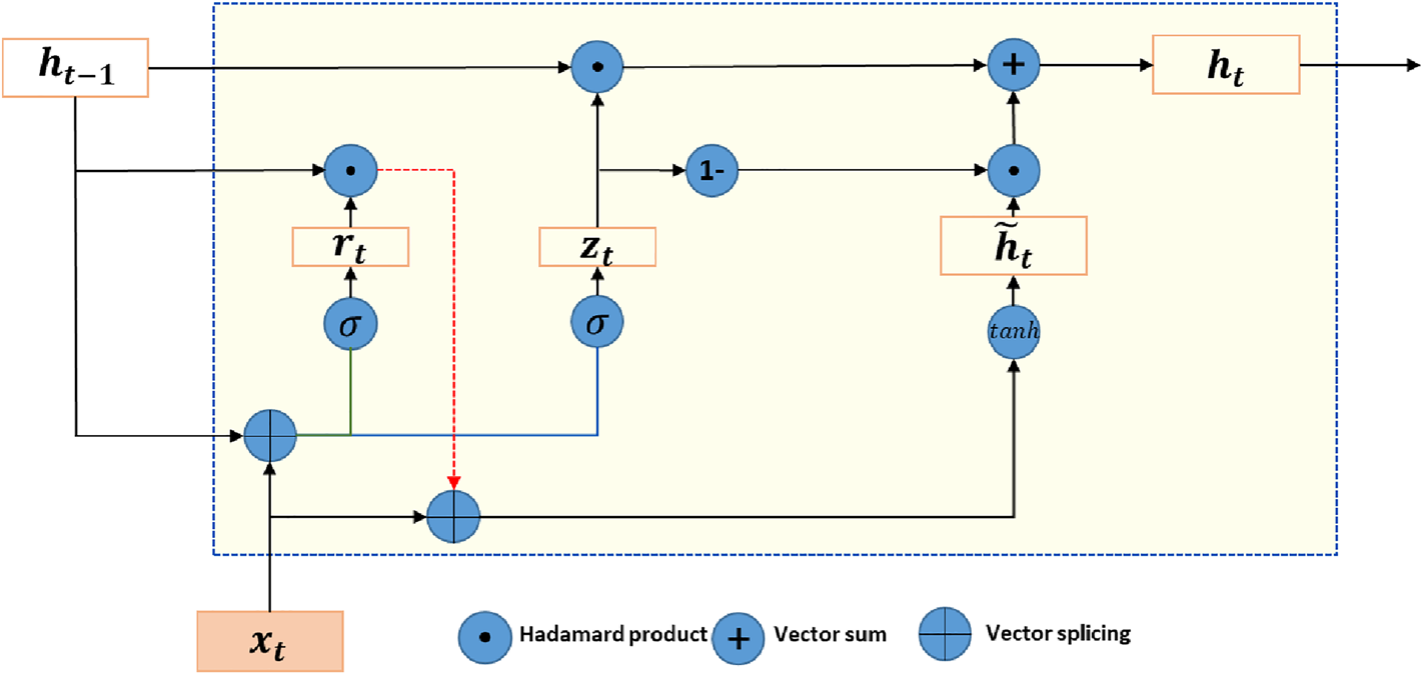
Recurrent unit structure of the GRU network.

The recurrent unit structure of the GRU network is shown in Fig. 6.

Each unit of GRU is calculated as follows:

7 $${z}_{t}= \sigma ({W}_{z}{x}_{t}+{U}_{z}{h}_{t-1}+{b}_{z})$$

8 $${r}_{t}= \sigma ({W}_{r}{x}_{t}+{U}_{r}{h}_{t-1}+{b}_{r})$$

9 $${\widetilde{h}}_{t}=tanh({W}_{h}{x}_{t}+{U}_{h}\left({r}_{t}\odot {h}_{t-1}\right)+{b}_{h})$$

10 $${h}_{t}={z}_{t}\odot {h}_{t-1}+\left(1-{z}_{t}\right)\odot {\widetilde{h}}_{t}$$

In the above formula, ${z}_{t}$ represents the update gate, which controls how much information is retained from the previous state ${h}_{t-1}$ (without nonlinear transformation) when calculating the current state ${h}_{t}$. Meanwhile, it also controls how much information will be accepted by ${h}_{t}$ from the candidate states ${\widetilde{h}}_{t}$. ${r}_{t}$ represents the reset gate, which is used to ensure whether the calculation of the candidate state ${\widetilde{h}}_{t}$ depends on the previous state ${h}_{t-1}$. $\upsigma$ is the standard sigmoid activation function; $tanh(\cdot )$ is the hyperbolic tangent activation function; and $\odot$ indicates the Hadamard product. The weight matrices of the update gate, reset gate, and ${\widetilde{h}}_{t}$ calculation layer are expressed as ${W}_{z},{W}_{r},{W}_{h}$; the coefficient matrices of the update gate, reset gate, and ${\widetilde{h}}_{t}$ calculation layer are expressed as ${U}_{z},{U}_{r},{U}_{h}$; and the offset vectors of the update gate, reset gate, and ${\widetilde{h}}_{t}$ calculation layer are expressed as ${b}_{z},{b}_{r},{b}_{h}$.

**Figure 7:**
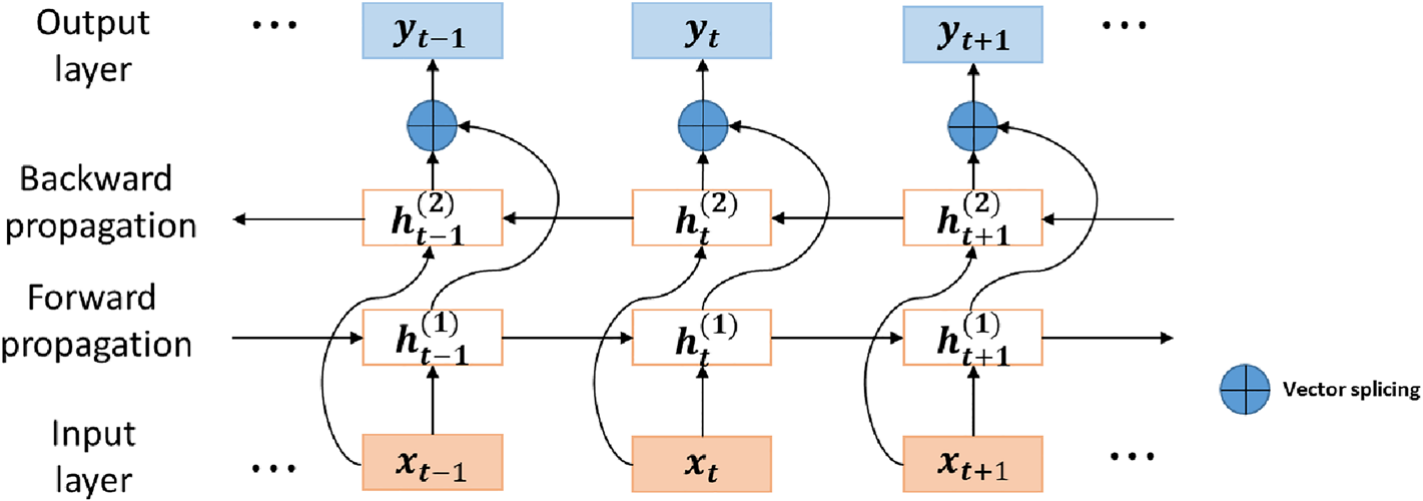
The structure of BiGRU.

A bidirectional gated recurrent unit network^37^ is a combination of two GRUs whose information propagating directions are reversed, and it has independent parameters in each, which makes it able to fit both forward and backward data at first and then join up the results from two directions. BiGRU can capture sequence patterns that may be ignored by unidirectional GRU. The structure of BiGRU is shown in Fig. 7.

Taking the BiGRU’s forward hidden state vector at time $t$ as ${h}_{t}^{(1)}$ and taking the BiGRU’s backward hidden state vector at time $t$ as ${h}_{t}^{(2)}$, $\upsigma$ indicates the standard sigmoid activation function, and $\oplus$ indicates a vector splicing operation. We can calculate the output ${y}_{t}$ of a BiGRU network as follows:

11 $${h}_{t}={h}_{t}^{(1)}\oplus {h}_{t}^{(2)}$$

12 $${y}_{t}=\sigma ({h}_{t} )$$

#### CNN-BiGRU prediction model

**Figure 8:**
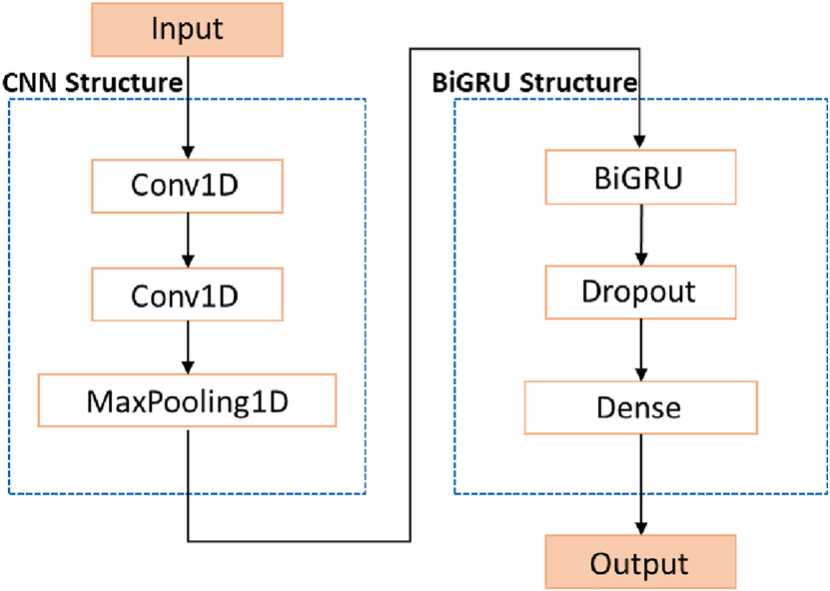
The structure of CNN-BiGRU.

Because CNN has significant advantages in extracting useful features from a picture or a sequence and BiGRU is good at processing time series, we combine CNN and BiGRU to build the CNN-BiGRU model. The model can be mainly divided into an input layer, a convolution layer, a BiGRU network layer, a dropout layer, a fully connected layer and an output layer. The CNN layer and BiGRU layer are the core structures of the model. The function of the dropout layer is to avoid model overfitting. The CNN layer consists of two one-dimensional convolution (Conv1D) layers and a one-dimensional maximum pooling (MaxPooling1D) layer. The input of BiGRU is the output sequence of the CNN layer, and the BiGRU network is set as a one-hidden-layer structure. The structure of the CNN-BiGRU combination model is shown in Fig. 8.

### Variable weight combination model

When we analyze and predict relatively stationary tide level time series, LightGBM can perform well. However, due to environmental factors such as air pressure, wind force and terrain in reality, most tide level observation sequences are sometimes not relatively stationary, which requires that our tide level prediction model be reasonably able to "extrapolate" based on the sample observations, that is, be capable of generating values that are not in the sample. LightGBM is a tree-based model, which leads to our prediction results being between the maximum and minimum values of sequences. Therefore, LightGBM will not be able to accurately predict the situation or tidal change trend that did not appear in previous observations. However, the CNN-BiGRU model, which is a kind of neural network, has no such problem in theory and will be able to find the trend information that may be hidden in the tide level series. Therefore, we consider providing an appropriate weight for a single base model to build a combination model to improve the accuracy of the tide level prediction task.

#### Principle of the residual weight combination model and improved variable weight combination model

To improve the prediction accuracy of the combination model, a simple and effective idea is to determine the base models’ weights in the combination model according to the error between the prediction value and the real value. This method is also called the residual weight method, and its calculation formulas for determining the weights are:

13 $$g\left({x}_{t}\right)= \sum_{i=1}^{m}{\omega }_{i}\left(t-1\right){f}_{i}({x}_{t})$$

14 $${\omega }_{i}\left(t-1\right)=\frac{\frac{1}{\overline{{\varphi }_{i}}\left(t-1\right)}}{\sum_{i=1}^{m}\frac{1}{\overline{{\varphi }_{i}}\left(t-1\right)}}$$

15 $$\sum_{i=1}^{m}{\omega }_{i}\left(t-1\right)=1,{\omega }_{i}\left(t-1\right)\ge 0$$

where ${\omega }_{i}\left(t-1\right)$ denotes the weight of the $i$ th model at the moment $t-1$, ${f}_{i}\left({x}_{t}\right)$ denotes the prediction value of the $i$ th model at the moment $t$, $g\left({x}_{t}\right)$ denotes the prediction value of the combination model at the moment $t$, and $\overline{{\varphi }_{i}}\left(t-1\right)$ is the square sum of the predictive errors of the $i$ th model at the moment $t-1$.

Our LightGBM-CNN-BiGRU (combination model) is based on the improved residual weight method. We call it the variable weight combination model. We use the weights calculated by formula (9) and formula (11) to calculate a series of new weights. The new weights from formula (11) will take the residual weight changes in $d$ time steps into consideration by averaging the old weights in $d$ time steps to improve the stability of the residual weight method.

16 $${\omega }_{j}\left(t\right)=\frac{1}{d}\sum_{k=1}^{d}{\omega }_{i}\left(t-k\right)\left(d=4\right)$$

After obtaining a series of weights through formula (9) and formula (11), we take the absolute value of the error between the prediction value and the true value of each combination model at the moment of $t$ as ${\delta }_{i,t}$ and ${\delta }_{j,t}$, respectively:

17 $${\delta }_{i,t}=\mid \sum_{i=1}^{m}{\omega }_{i}\left(t\right){f}_{i}\left({x}_{t}\right)-{y}_{t}\mid $$

18 $${\delta }_{j,t}=\mid \sum_{i=1}^{m}{\omega }_{j}\left(t\right){f}_{i}\left({x}_{t}\right)-{y}_{t}\mid $$

Comparing ${\delta }_{i,t}$ and ${\delta }_{j,t}$, if ${\delta }_{i,t}>{\delta }_{j,t}$, the combination model uses the new weight ${\omega }_{j}\left(t\right)$ in place of the original weight ${\omega }_{i}\left(t\right)$. Otherwise, the weight of the combination model remains unchanged.

#### Parameter optimization of the combination model

Because the LightGBM-CNN-BiGRU (combination model) is a variable weight combination of the prediction results from two base models, the performance of the combination model can be directly improved by separately optimizing the super parameters of the two base models. We mainly use the grid search algorithm and K-fold cross validation method to optimize the parameters. The grid search algorithm is a method to improve the performance of a certain model by iterating over a given set of parameters. With the help of the K-fold cross validation method, we can calculate the performance score of the LightGBM model on the training set and easily optimize its superparameters. The final parameters of the LightGBM model are set to num_leaves = 26, learning_rate = 0.05, and n_estimators = 46.

For the CNN-BiGRU network, we mainly improve the prediction accuracy of the model by adjusting the size and number of hidden layers in the BiGRU structure and prevent the model from overfitting by changing the dropout ratio and tracking the validation loss of the network while training.

#### The LightGBM and CNN-BiGRU variable weight combination model

**Figure 9:**
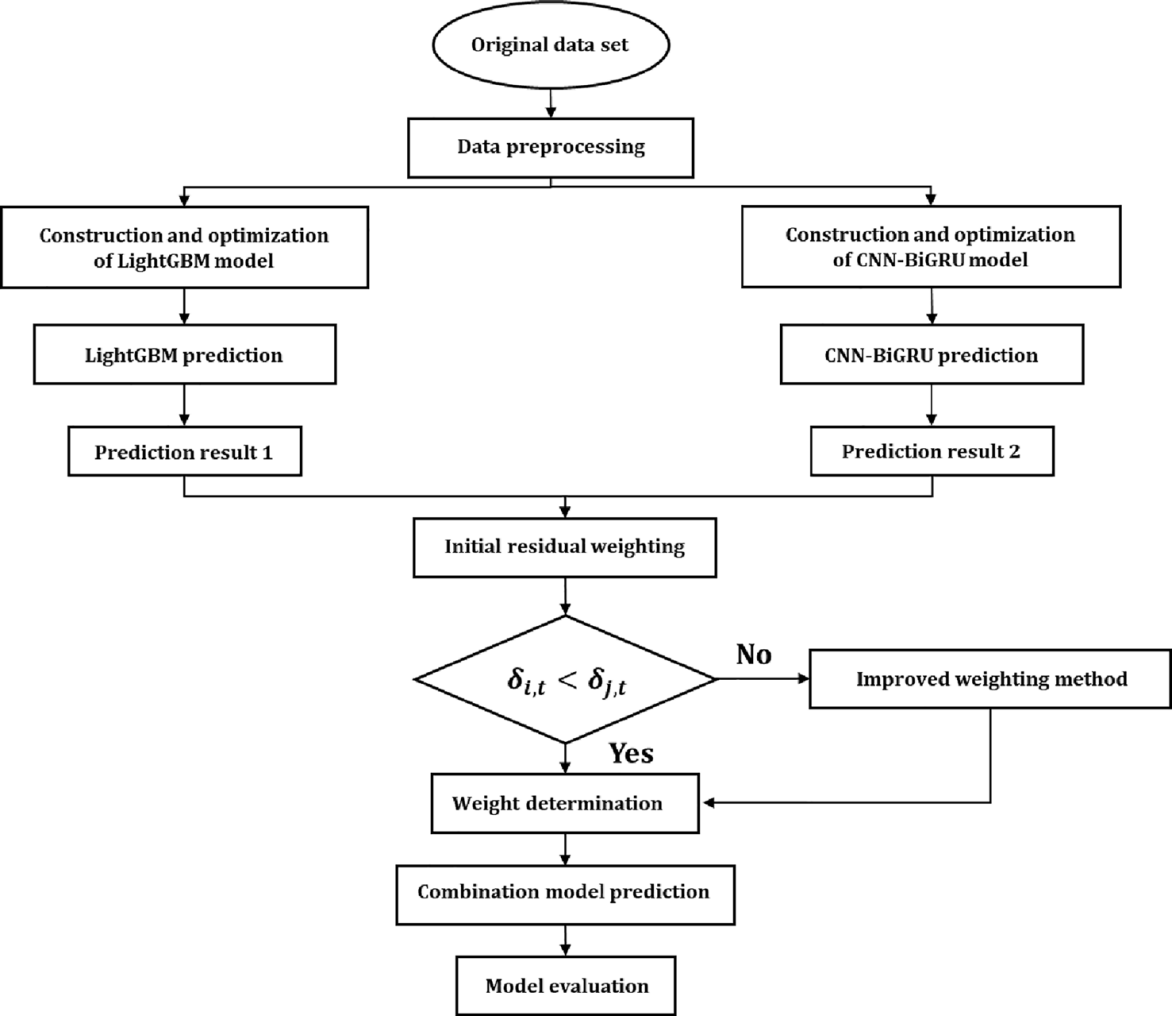
Prediction flow of the LightGBM-CNN-BiGRU variable weight combination model.

The workflow of our tide level prediction model is shown in Fig. 9. It mainly includes data preprocessing; training, optimization and prediction of the base models; construction of a variable weight combination prediction model; and evaluation and analysis of the combination model’s performance.

1. Data preprocessing: The quality of the data directly determines the upper limit of the prediction and generalization ability of a certain machine learning model. Standard, clean and continuous data are conducive to model training. The data used in this study are from the Irish National Tide Gauge Network, and all of them are subject to quality control. We filled in a small number of missing values and normalized the data to speed up the model training.
2. Construction and optimization of base models: We divide the dataset into a training set, a validation set and a test set according to the proportion of 7:1:2 and train the LightGBM model and CNN-BiGRU model with data on the training set. We optimize the parameters and monitor whether the model has been overfitted by tracking the validation loss of the network while training. Finally, we put the data into two base models for training and then obtain the prediction results of a single base model.
3. Construction of the variable weight combination model. Based on the prediction results of two single base models obtained in step (2), we calculate the weight of each base model according to the principle of the improved variable weight combination method and then obtain the prediction results of the variable weight combination model.
4. Model evaluation and analysis: According to the indexes of the model evaluation, the variable weight combination model is compared with other basic models to analyze its prediction performance after being improved.

## Results

To evaluate the performance of the LightGBM-CNN-BiGRU (combination model) we proposed on the task of short-term tidal level prediction, we took the tidal observations of the Howth Harbor site in the first quarter of 2017 as the sample, used a sliding data window ($T=10$) to build input data, and let the models predict the tide level of the next time step.

### Evaluation indicator

We use the root mean square error (RMSE) and mean absolute error (MAE) as the evaluation criteria of model prediction performance. The calculation formula is:

19 $$RMSE=\sqrt{\frac{1}{m}\sum_{k=1}^{m}{\left({y}_{k}-\widehat{{y}_{k}}\right)}^{2}}$$

20 $$MAE=\frac{1}{m}\sum_{k=1}^{m}\left|{y}_{k}-\widehat{{y}_{k}}\right|$$

where $m$ is the number of test samples and ${y}_{k}$ and $\widehat{{y}_{k}}$ represent the observed value and prediction value of the tide level, respectively. If the loss function of the model is small, the prediction value will be closer to the observed value, and the calculation of RMSE and MAE will also be smaller.

### A comparative analysis of BiLSTM and BiGRU

When building tidal prediction models, previous scholars mostly used LSTM or BiLSTM as the constituent unit of recurrent neural networks^19–21^. In many industrial practices, the prediction performances of LSTM and GRU are considered to have no significant difference. Based on the existing data, we designed a simple comparative experiment to show that the BiGRU unit can achieve higher prediction accuracy than BiLSTM in the tide level prediction task.

**Figure 10:**
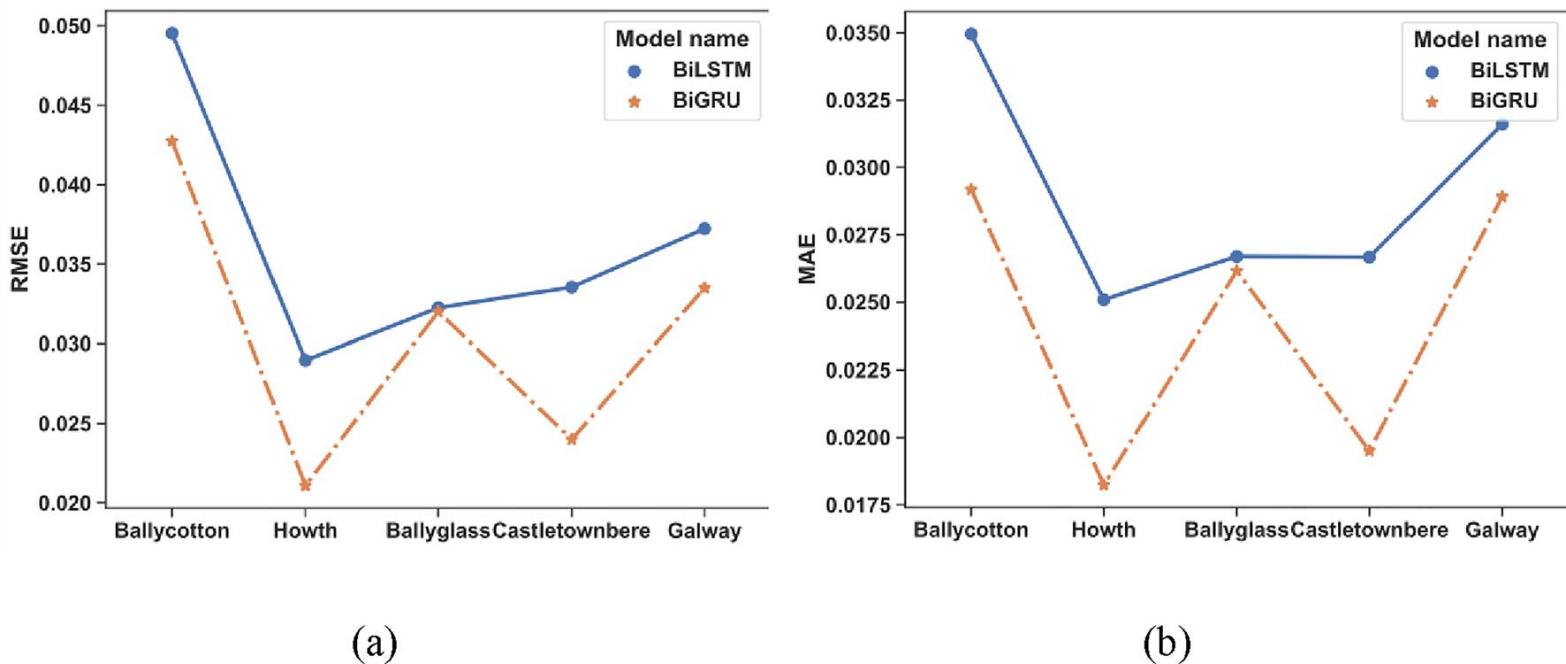
Comparison of prediction effects between BiLSTM and BiGRU on different sites.

After many experiments, we found that the network structure of single-layer BiLSTM or single-layer BiGRU is sufficient for tide level prediction. Therefore, we control the number of training epochs of the two models to be the same and the size of their hidden layer to be similar and then carry out the prediction on the tidal observations from five ports. The prediction accuracy of the two kinds of networks is shown in Fig. 10a,b. Through the experimental results, it can be found that using GRU as the constituent unit can achieve better results in the tidal prediction task when the structure of the recurrent neural network is almost the same. Therefore, we choose the BiGRU structure to participate in the construction of the CNN-BiGRU base model rather than the traditional BiLSTM structure.

### Prediction analysis and comparison of combined models

**Figure 11:**
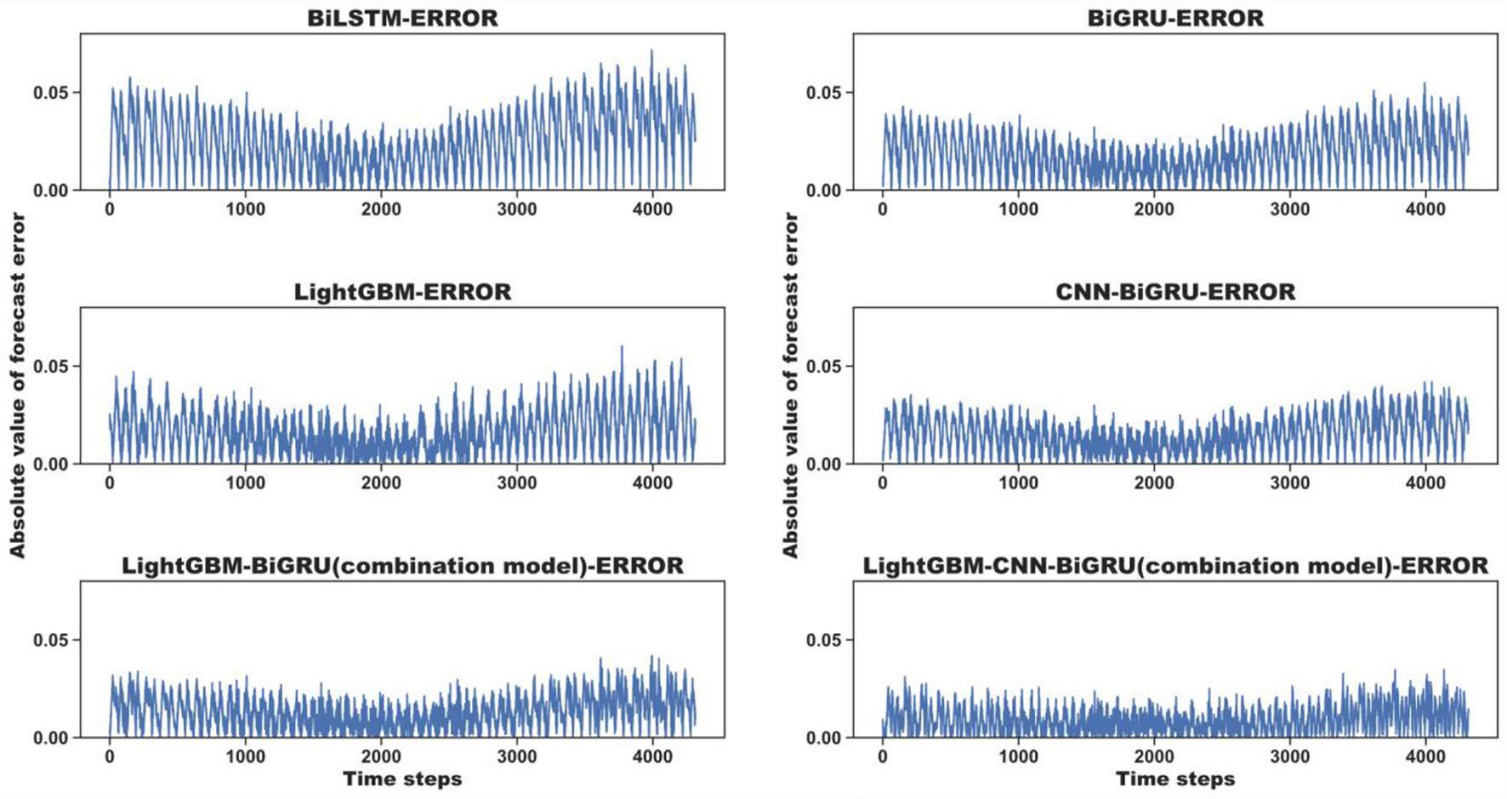
Prediction error of each model.

To verify the good prediction ability of our variable weight combination model based on LightGBM and CNN-BiGRU, we use the observations of the Howth Harbor site to construct the tide prediction model we proposed and select the simple model BiGRU, the base model LightGBM, CNN-BiGRU, and another variable weight combination model called LightGBM-BiGRU (combination model) as our comparison models. The comparison of absolute prediction errors between different models is shown in Fig. 11. As seen from Fig. 11, the prediction error of the LightGBM-CNN-BiGRU (combination model) is the smallest, and its prediction accuracy is significantly higher than that of the simple BiLSTM and BiGRU models and the LightGBM and CNN-BiGRU base models.

Table 2 shows the RMSE and MAE values of each model on the test set. After analysis, compared with the second-best model LightGBM-BiGRU (combination model), the RMSE and MAE values of LightGBM-CNN-BiGRU (combination model) are reduced by 26.6% and 29.8%, respectively. To some extent, this shows that using a CNN layer for preliminary information extraction can improve the prediction accuracy of the BiGRU model. Meanwhile, compared with the base model LightGBM, the RMSE and MAE values of the combination model are reduced by 43.2% and 44.7%, respectively; compared with the base model CNN-BiGRU, the RMSE and MAE values of the combination model are reduced by 35.3% and 39.1%, respectively. The model using the variable weight combination method has a higher prediction accuracy.

**Table 2:** Prediction effects of different models on the Howth Harbor site.

Model	RMSE/m	MAE/m
BiLSTM	0.137	0.119
BiGRU	0.100	0.086
LightGBM	0.093	0.077
CNN-BiGRU	0.081	0.070
LightGBM–BiGRU (combination model)	0.072	0.061
LightGBM–CNN-BiGRU (combination model)	0.052	0.043

### Model generality analysis

Due to environmental factors such as air pressure, wind force and topography, the tidal observations obtained in different areas should have a degree of pattern differences. To further determine whether the LightGBM-CNN-BiGRU (combination model) has good universality and generalization ability for tide level prediction, under the same simulation conditions, we selected the data of the other four sites on the Irish coastline for prediction experiments. The subsequent prediction results are shown in Table 3.

**Table 3:** Comparison of the prediction accuracy between the combination model and its base model.

Site	RMSE/m			MAE/m		
	LightGBM	CNN-BiGRU	LightGBM–CNN-BiGRU	LightGBM	CNN-BiGRU	LightGBM–CNN-BiGRU
			(combination model)			(combination model)
Howth Harbor	0.093	0.081	0.052	0.077	0.070	0.043
Ballycotton Harbor	0.143	0.170	0.116	0.097	0.062	0.040
Ballyglass Harbor	0.083	0.035	0.029	0.067	0.028	0.023
Castletownbere Port	0.084	0.066	0.054	0.068	0.051	0.042
Galway Port	0.107	0.044	0.037	0.085	0.036	0.030

It is not difficult to find that the RMSE and MAE indexes of the variable weight combination model (LightGBM—CNN-BiGRU (combination model)) are better than those of the single base model. After calculation, compared with the single base model, the RMSE of the variable weight combination model is reduced by at least 16.2%, and the MAE is reduced by at least 16.7%. To conclude, the prediction results of the variable weight combination model based on LightGBM and CNN-BiGRU are more accurate, and the universality and generalization ability of the model have been verified.

## Discussion and conclusion

Accurate tidal prediction is of great significance for human activities in coastal areas. The traditional harmonic analysis method for tide level prediction needs to take the local hydrological, meteorological and geographical conditions into consideration and depends on a large amount of tide observation data. Even still, there is a large prediction error when using it. Machine learning models, represented by LSTM, XGBoost and LightGBM, with their wide applicability and strong fitting ability, have been proven by many studies to be capable of predicting tide levels accurately. Based on previous research, this paper proposes a variable weight combination model based on LightGBM and CNN-BiGRU. It combines the predictions of LightGBM and CNN-BiGRU models with variable weights to further improve the accuracy of tide prediction and to compensate for the lack of "extrapolation" of LightGBM to a certain extent.

**Figure 12:**
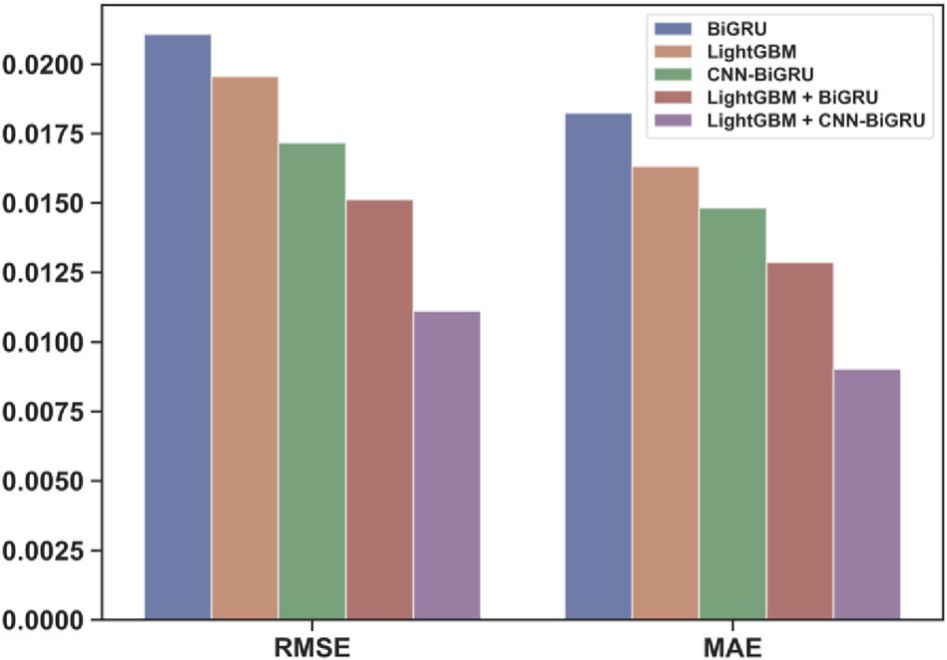
Prediction results of different models on the Howth Harbor site.

We use the data of five stations from the real-time tide monitoring network INTGN operated by the Irish Marine Institute for all model construction and analysis and decide to predict the Howth Harbor’s tide level in the next time step by using the observed values within 1 h, with the purpose of verifying the good short-term tide level prediction ability of our combination model. As shown in Fig. 12, our combination model enjoys very high prediction accuracy. After analysis, compared with the base model LightGBM, the RMSE and MAE values of the LightGBM—CNN-BiGRU (combination model) are reduced by 43.2% and 44.7%, respectively. Compared with the other base model CNN-BiGRU, the RMSE and MAE values of the LightGBM-CNN BiGRU (combination model) are reduced by 35.3% and 39.1%, respectively. We believe that the reason why the model can achieve such a good performance is that LightGBM can fit the nonlinear characteristics of the data well, while CNN-BiGRU is good at mining the temporal characteristics of the data. The combined model combines the advantages of the two base models through the improved residual weight method. We also analyzed the generality of the combination model. Based on Howth Harbor’s observations, we selected data from the other four stations on the Irish coastline to carry out further prediction experiments. After calculation, compared with the single base models, the RMSE of the variable weight combination model based on LightGBM and CNN-BiGRU is reduced by at least 16.2%, while the MAE is reduced by at least 16.7%. The good prediction performance and generalization ability of the model have been further verified.

In addition, we can conclude from the example analysis that using GRU as the constituent unit can achieve better results in the tidal prediction task when the structure of the recurrent neural network is almost the same. While using the BiGRU network for tide level prediction, adding a CNN module to the network at first for information extraction can effectively improve the accuracy of model prediction. Moreover, the variable weight combination method has good universality to some extent. Both LightGBM-BiGRU (combination model) and LightGBM-CNN-BiGRU (combination model) predict more accurately than their respective base models.

Indeed, our research still has many areas that can be improved. Due to the limited data collection, we only carried out prediction research on tide observations along the coast of Ireland. If we can collect tidal observation data from other places of the world in the future, we will be able to further verify the good prediction performance and the strong generalization ability of the variable weight combination model based on LightGBM and CNN-BiGRU. We will also try the multistep prediction ability of the combination model to realize tide prediction in a longer time. In short, our research shows that the LightGBM-CNN-BiGRU (combination model) we proposed can achieve higher prediction accuracy than traditional tide prediction models such as BiLSTM, and the variable weight combination method can enable the combination model to achieve better prediction performance than its single base models. Our experiment also shows that the combination model can reduce the absolute error to approximately 0.03 m with only one quarter of the tide observations, and the construction of the model is not complicated, so it deserves to be used in practice.

## Data Availability

The datasets can be downloaded on this website: http://data.marine.ie/geonetwork/srv/eng/catalog.search#/metadata/ie.marine.data:dataset.2774.

## Code availability

Reproduce our results through cloning the code from the GitHub repository: suye0620/TidalPrediction.

## References

[1] Darwin GH, Turner HHI (1886). On the correction to the equilibrium theory of tides for the continents [J]. Proc. R. Soc. Lond..

[2] Doodson AT (1921). The harmonic development of the tide-generating potential [J]. Proc. R. Soc. Lond. Ser. A.

[3] Doodson AT (1924). Perturbations of harmonic tidal constants [J]. Proc. R. Soc. Lond. Ser. A.

[4] Doodson ATVI (1928). The analysis of tidal observations [J]. Philos. Trans. R. Soc. Lond. Ser. A.

[5] Kukulka, T., & Jay, D. A. Impacts of Columbia River discharge on salmonid habitat: 1. A nonstationary fluvial tide model [J]. *J. Geophys. Res. Oceans* **108**(C9) (2003).

[6] Kukulka, T., & Jay, D. A. Impacts of Columbia River discharge on salmonid habitat: 2 Changes in shallow-water habitat [J]. *J. Geophys. Res.: Oceans* **108**(C9) (2003).

[7] Jin GZ, Pan HD, Zhang QL (2018). Determination of harmonic parameters with temporal variations: An enhanced harmonic analysis algorithm and application to internal tidal currents in the South China Sea [J]. J. Atmos. Oceanic Tech..

[8] Pan HD, Guo Z, Lv XQ (2017). Inversion of tidal open boundary conditions of the M 2 constituent in the Bohai and Yellow Seas [J]. J. Atmos. Oceanic Tech..

[9] Fan, W. Data assimilation and numerical study on a marine ecosystem model [D]; Ocean University of China (2009).

[10] Li, X. Y. Optimization of the spatio-temporal parameters in a dynamical marine ecosystem model based on the adjoint assimilation [D]; Ocean University of China (2013).

[11] Wang, C. H. Numerical study and application of a marine ecosystem dynamical model with adjoint assimilation method [D]; Ocean University of China (2013).

[12] Matte P, Jay DA, Zaron ED (2013). Adaptation of classical tidal harmonic analysis to nonstationary tides, with application to river tides [J]. J. Atmos. Oceanic Tech..

[13] Pan H, Lv X, Wang Y (2018). Exploration of tidal-fluvial interaction in the Columbia River Estuary using S_TIDE [J]. J. Geophys. Res. Oceans.

[14] Xianqing L, Haidong P, Yuzhe W (2021). Review and prospect of tidal harmonic analysis methods [J]. Mar. Sci..

[15] Tsai C-P, Lee T-L (1999). Back-propagation neural network in tidal-level forecasting [J]. J. Waterw. Port Coast. Ocean Eng..

[16] Tsai C-P, Lin C, Shen J-N (2002). Neural network for wave forecasting among multi-stations [J]. Ocean Eng..

[17] Zhang ZG, Yin JC, Liu C (2017). SAPSO-BP network in tidal level prediction of port [J]. Port Waterway Eng..

[18] Zhang ZG, Yin JC, Liu C (2018). Modular real-time tidal level prediction based on Grey-GMDH [J]. Period. Ocean Univ. China.

[19] Zhu GQ (2020). Research on short-term tide forecast based on Bi-LSTM recurrent neural network [J]. Int. J. Soc. Sci. Educ. Res..

[20] Yang C-H, Wu C-H, Hsieh C-M (2020). Long short-term memory recurrent neural network for tidal level forecasting [J]. IEEE Access.

[21] Huang DM, Wang C, Hu AD (2021). Tide level prediction for tidal power station based on CNN-BiLSTM network model [J]. Water Power.

[22] Giles CL, Kuhn GM, Williams RJ (1994). Dynamic recurrent neural networks: Theory and applications [J]. IEEE Trans. Neural Netw..

[23] Ke, G., Meng, Q., & Finley, T., *et al.* Lightgbm: A highly efficient gradient boosting decision tree [J]. Adv. Neural Inf. Process. Syst. **30** (2017).

[24] Chen, T., & Guestrin, C. Xgboost: A scalable tree boosting system; proceedings of the Proceedings of the 22nd acm sigkdd international conference on knowledge discovery and data mining, F [C] (2016).

[25] Gumus, M., & Kiran, M. S. Crude oil price forecasting using XGBoost; proceedings of the 2017 International conference on computer science and engineering (UBMK), F, [C]. IEEE (2017).

[26] Sun XL, Liu MX, Sima ZQ (2020). A novel cryptocurrency price trend forecasting model based on LightGBM [J]. Financ. Res. Lett..

[27] Elsayed, S., Thyssens, D., & Rashed, A, *et al.* Do we really need deep learning models for time series forecasting? [J]. arXiv preprint arXiv:210102118 (2021).

[28] Zhang XJ, Liu F (2020). Gas concentration prediction in coal mines based on wavelet noise reduction and recurrent neural networks [J]. Coal Technol..

[29] Han TT, Wu SY (2014). Gas concentration prediction based on Markov residual correction [J]. Ind Min. Autom..

[30] Friedman, J. H. Greedy function approximation: a gradient boosting machine [J]. *Ann. Stat.* 1189–1232 (2001).

[31] G D T. An experimental comparison of three methods for constructing ensembles of decision trees: bagging, boosting, and randomization [J]. *Mach. Learn.* **40** (2000).

[32] Rawat W, Wang Z (2017). Deep convolutional neural networks for image classification: A comprehensive review [J]. Neural Comput..

[33] Gu J, Wang Z, Kuen J (2018). Recent advances in convolutional neural networks [J]. Pattern Recogn..

[34] Bai, S. J., Kolter, J. Z., & Koltun, V. An empirical evaluation of generic convolutional and recurrent networks for sequence modeling [J]. arXiv preprint arXiv:180301271 (2018).

[35] Hochreiter S, Schmidhuber J (1997). Long short-term memory [J]. Neural Comput..

[36] Cho, K., Van Merriënboer, B., Gulcehre, C.,* et al*. Learning phrase representations using RNN encoder-decoder for statistical machine translation [J]. arXiv preprint arXiv:14061078 (2014).

[37] Schuster M, Paliwal KK (1997). Bidirectional recurrent neural networks [J]. IEEE Trans. Signal Process..

